# Combination of metformin/efavirenz/fluoxetine exhibits profound anticancer activity via a cancer cell-specific ROS amplification

**DOI:** 10.1080/15384047.2022.2161803

**Published:** 2023-01-01

**Authors:** Beom-Goo Kang, Madhuri Shende, Gozde Inci, Soo-Hyun Park, Jun-Sub Jung, Set Byeol Kim, Jeong Hoon Kim, Young Won Mo, Ji-Hyeon Seo, Jing-Hui Feng, Sung-Chan Kim, Soon Sung Lim, Hong-Won Suh, Jae-Yong Lee

**Affiliations:** a Department of Biochemistry, Institute of Cell Differentiation and Aging, College of Medicine, Hallym University, Chuncheon, Republic of Korea; b FrontBio Inc, Gangwon-do, Republic of Korea; c Department of Food Science and Nutrition, College of Natural Science, Hallym University, Chuncheon, Republic of Korea; d Department of Pharmacology, Institute of Natural Medicine, Hallym University, Chuncheon, Republic of Korea

**Keywords:** metformin/efavirenz/fluoxetine, anticancer activity, FOXO3a, MnSOD, cancer cell-specific, ROS amplification, mitochondrial complex I and III, apoptosis/necroptosis/autophagy

## Abstract

The possible anticancer activity of combination (M + E + F) of metformin (M), efavirenz (E), and fluoxetine (F) was investigated in normal HDF cells and HCT116 human colon cancer cells. Metformin increased cellular FOXO3a, p-FOXO3a, AMPK, p-AMPK, and MnSOD levels in HDFs but not in HCT116 cells. Cellular ATP level was decreased only in HDFs by metformin. Metformin increased ROS level only in HCT116 cells. Transfection of si-FOXO3a into HCT116 reversed the metformin-induced cellular ROS induction, indicating that FOXO3a/MnSOD is the key regulator for cellular ROS level. Viability readout with M, E, and F alone decreased slightly, but the combination of three drugs dramatically decreased cell survival in HCT116, A549, and SK-Hep-1 cancer cells but not in HDF cells. ROS levels in HCT116 cells were massively increased by M + E + F combination, but not in HDF cells. Cell cycle analysis showed that of M + E + F combination caused cell death only in HCT116 cells. The combination of M + E + F reduced synergistically mitochondrial membrane potential and mitochondrial electron transport chain complex I and III activities in HCT116 cells when compared with individual treatments. Western blot analysis indicated that DNA damage, apoptosis, autophagy, and necroptosis-realated factors increased in M + E + F-treated HCT116 cells. Oral administration with M + E + F combination for 3 weeks caused dramatic reductions in tumor volume and weight in HCT116 xenograft model of nude mice when compared with untreated ones. Our results suggest that M + E + F have profound anticancer activity both *in vitro* and *in vivo* via a cancer cell-specific ROS amplification (CASRA) through ROS-induced DNA damage, apoptosis, autophagy, and necroptosis.

## Introduction

Reactive oxygen species (ROS) are termed as highly reactive oxygen molecules that are a byproduct of metabolism^1^. ROS have been considered a therapeutic means of promoting cell death in cancer cells.^2–4^ As destructive ROS are to lipids, proteins, and DNA, they also play a role in regulation of survival and proliferation of the cell. Cancer cells already have elevated levels of ROS from increased rates of metabolism, which lead to an increase in their rates of genetic alteration. Such elevated levels of ROS can contribute to tumor promotion and progression for via increased DNA mutation and metabolism. As such, slight increases in ROS levels would not be enough to cause cancer cell death, and they need to be increased beyond a certain level to contribute to cancer cell death for therapy. Various strategies have attempted to achieve this. A dual generating chemotherapy and oxidative stress strategies, such as a cyclic diselenide of gemcitabine, has been proposed for cancer cell-specific cell death.^5^ In addition, ferroptosis inducers for a massive resultant ROS have also been proposed for this purpose.^6^ It is noted that exposure to various ROS amplifiers makes cancer cells more vulnerable than normal cells.^7,8^ In this area, we recently reported a cancer-cell-specific ROS amplification via a combination of mild inhibitors of mitochondrial electron transport chain complex I/III, metformin, and the ROS amplifier, apigenin.^9^

Metformin, a biguanide, interacts and inhibits mitochondrial electron transport chain complex I and III. This causes the induction of cellular ROS increase by a mild leakage of electron transport, resulting in a mild ATP production decrease. In the metformin pathway, reduced cellular ATP levels usually cause activation of AMP-activated protein kinase (AMPK), which is followed by activation of the transcription factor, forkhead box O3a (FOXO3a), through its direct phosphorylation by AMPK. This is significant as FOXO3a plays an important role in regulation of cell growth and death, ROS detoxification, glucose metabolism, and longevity.^10,11^ For ROS detoxification, FOXO3a protein reduces oxidative stress by increasing the cellular levels of antioxidant enzymes, catalase, an enzyme for removal of hydrogen peroxide, and MnSOD (manganese superoxide dismutase), an enzyme for removal of superoxide.^10,12,13^

Cancer cells generate 2 moles of ATP per mole of glucose via aerobic glycolysis and lactate fermentation, also known as “the Warburg effect,” while ATP production through mitochondrial oxidative phosphorylation is mostly not preferred in cancer cells.^14^ By comparison, for normal cells having dominant levels of oxidative phosphorylation, they produce 30 or 32 moles of ATP per mole of glucose when oxygen is supplied sufficiently. This difference between normal and cancer cells results in different responses to metformin treatment. Metformin reduces cellular ATP levels and activates AMPK (as p-AMPK at Thr172) and FOXO3a (as p-FOXO3a at Ser413) only in normal cells. FOXO3a then transcriptionally activates MnSOD and catalase.^10^ These two antioxidant enzymes remove cellular ROS in normal cells; however, ROS levels remain high in cancer cells.^9^ Metformin has been reported to reduce cellular ROS levels in normal cell including mouse embryonic fibroblast cells and immune cells.^15^ However, metformin has been controversially reported to reduce^16^ or to increase^17^ cellular ROS level in cancer cells. No report has carefully compared the difference of ROS production in normal cells and cancer cells.

Several reports have demonstrated that metformin possesses an anticancer-effects.^18–21^ It has been shown that metformin is effective in producing apoptosis in several tumor types.^22,23^ In addition, the combination of metformin with certain anticancer drugs such as doxorubicin,^24^ trametinib,^25^ and cisplatin^26^ displays an effective anticancer activity in tumor xenografted animal models. After we found that metformin differentiated in ROS production between normal and cancer cells, we have performed screening of drugs that showed a strong interaction with metformin for anti-cancer activity via ROS amplification. We found that efavirenz or fluoxetine showed an enhancing effect on ROS production and anticancer activity when interacting with metformin, respectively.

Efavirenz, which is an inhibitor of non-nucleotide reverse transcriptase, has been used as an antiviral drug for human immunodeficiency virus. However, efavirenz showed severe side effects including lipid and metabolic disorders, psychiatric symptoms, and hepatotoxicity.^27–29^ Inhibition of mitochondrial function was turned out to be responsible for these side effects. It has been reported that efavirenz inhibits the mitochondrial membrane potential and electron transport chain complex I. Thereby efavirenz increases cellular superoxide production and oxidative injury.^30,31^ The cellular toxicity of efavirenz was reported to provide antitumor effect in various cancer cells including pancreatic cancer,^32^ colorectal carcinoma, and Jurkat (acute T-cell leukemia) cells.^33^ Fluoxetine is currently being used for treatment of depression. Fluoxetine has been shown to cause ROS amplification primarily through the mitochondrial pathway.^34^ Furthermore, it has been reported that fluoxetine possesses anticancer activity both *in vitro* and *in vivo*.^35,36^ In addition, fluoxetine enhances the anticancer activity of certain chemotherapeutic anticancer drugs such as gemcitabine and paclitaxel.^37–39^

Colon cancer is the third most common malignant tumor in humans. Chemotherapy has been used mainly to treat colon cancer. However, due to drug resistance, the effect of chemotherapy is still not satisfactory. The resistance to drug appears to be due to the presence of highly metastatic cancer stem cells and the tumor microenvironment. Since the targeted therapy is not available and development of immunotherapy has just started, colon cancer has been one of the hardest cancers to treat.^40^

In this study, we attempted a combination of metformin/efavirenz/fluoxetine to demonstrate an excellent anticancer activity in cultured cells and also in a xenograft mouse model of human colon cancer cells via cancer-cell-specific ROS amplification (CASRA). We also suggest a possible mechanism for the synergistic anticancer activity seen with this combination over single-drug treatments by measuring inhibition of mitochondrial electron chain complexes.

## Results

### Metformin induces cancer cell-specific ROS increase via the AMPK/FOXO3a/MnSOD pathway

We compared AMPK activation by metformin in cancer and normal cells along with activation of FOXO3a, as the latter is downstream of AMPK. HCT116 and HDF cells were treated with metformin (50 µM, 500 µM 5 mM) for 24 hr and immunofluorescence analysis of the treated cells with anti p-AMPK and p-FOXO3a (Ser413) antibodies.

**Figure 1.**
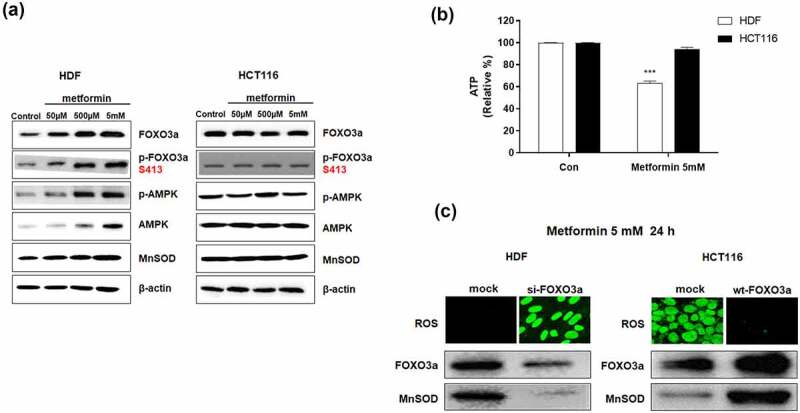
Effect of metformin on FOXO3a, p-FOXO3a, AMPK, p-AMPK, MnSOD, ATP, and ROS levels in HCT116 and HDF cells. (a) HCT116 and HDF cells were treated with metformin (0.05, 0.5, 5 mM) for 24 h. The levels of AMPK, p-AMPK (Thr172), FOXO3a, p-FOXO3a (Ser413) and MnSOD proteins were measured in metformin-treated HCT116 and HDF cells by western blot analysis. (b) Cellular ATP levels were measured in HCT116 and HDF cells after metformin (5 mM) treatment for 24 h. (c) Changes in cellular ROS levels were measured after 24 h post transfection with si-FOXO3a RNA into metformin-treated HDF cells or wt-FOXO3a plasmids into metformin-treated HCT116 cells. Cellular ROS levels were measured by CellROX Green staining. Protein levels of FOXO3a and MnSOD were measured by western blot analysis. Statistical significance is indicated as ***p < .001. Figure 1. Metformin treatment increased the protein levels of AMPK, p-AMPK, FOXO3a, p-FOXO3a and MnSOD only in HDF normal cells but not in HCT116 colon cancer cells. ATP decreased only in HDF normal cells but not in HCT116 colon cancer cells because normal cells produce ATP via mitochondrial oxidative phosphorylation and inhibition of mitochondria by metformin decreased ATP production. Cancer cells did not show ATP decrease because they produce ATP mainly through lactate fermentation. Overexpression of si-FOXO3a in HDF normal cells reduced the protein levels of FOXO3a/MnSOD (a FOXO3a target gene) and increased cellular ROS (reactive oxygen species) level, while overexpression of wt-FOXO3a in HCT116 colon cancer cells increased the protein levels of FOXO3a /MnSOD and decreased cellular ROS (reactive oxygen species) level.

Activated FOXO3a (p-FOXO3a S413) increases the transcription of MnSOD. These are revealed in western blot analysis, with AMPK, p-AMPK, FOXO3a, p-FOXO3a, and MnSOD expression levels increasing only in HDF normal cells but not in HCT116 colon cancer cells (Figure 1a). Cellular ATP levels decreased by up to 40% with metformin treatment of HDF cells but were almost unchanged in HCT116 cells (Figure 1b). Metformin interferes with mitochondrial electron transport systems, causing a level of electron leakage to decrease ATP production in normal HDF cells.^41^ Although there is some electron leakage in cancer cell due to metformin, their cellular ATP levels were unchanged in HCT116 cells because ATP is mostly produced by aerobic glycolysis and lactate fermentation in cancer cells. The decreased cellular ATP levels in normal HDF cells indicate a decrease in cellular energy levels. This situation increases the levels of AMPK/p-AMPK with p-AMPK activating FOXO3a by direct phosphorylation of FOXO3a S413. Activated FOXO3a (p-FOXO3a S413) increases the transcription of MnSOD. These are revealed in western blot analysis, with AMPK, p-AMPK, FOXO3a, p-FOXO3a, and MnSOD expression levels increasing only in HDF normal cells but not in HCT116 colon cancer cells (Figure 1a). In addition, the cellular ROS levels were nearly undetectable in HDFs treated with metformin (Figure 1c) due to increased protein levels of MnSOD, a cellular ROS scavenger enzyme. However, cellular ROS levels abruptly increased in HCT116 cells post metformin treatment as there was no activation of the AMPK/FOXO3a/MnSOD pathway. To test whether FOXO3a is an important mediator of cancer cell-specific ROS increase by metformin, HDFs were treated by siFOXO3a RNA, and they were assayed for ROS changes. Cellular ROS levels were markedly increased by si-FOXO3a RNA treatment of HDFs, and the protein levels of FOXO3a and MnSOD were decreased in HDFs as expected. By comparison, transfection of HCT116 cells with wt-FOXO3a caused an increase in protein levels of FOXO3a and MnSOD and a dramatic decrease of their cellular ROS levels (Figure 1c), indicating that FOXO3a is an important mediator for cancer cell-specific ROS increase by metformin.

### Combination of metformin, efavirenz, and fluoxetine amplifies cellular ROS and induces cell death in HCT116 human colon cancer cells

**Figure 2.**
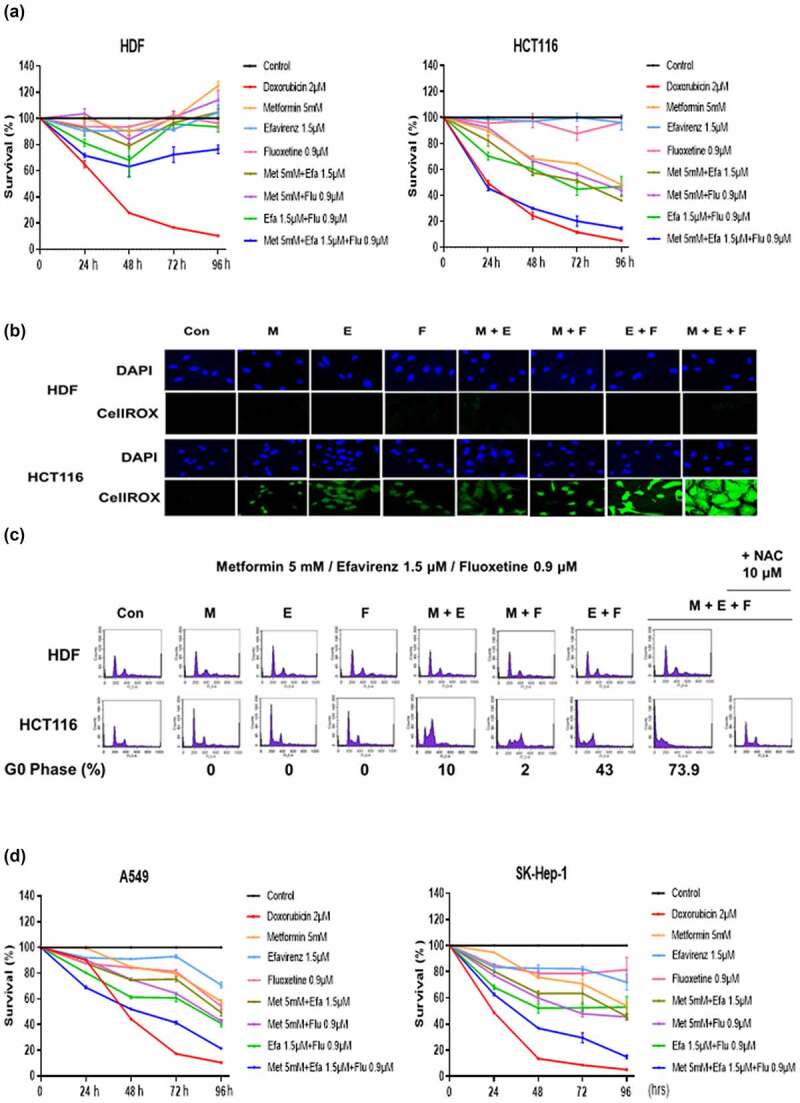
Changes in cell survival, cellular ROS levels, and cell death by the combination of metformin/efavirenz/fluoxetine in HDFs and HCT116 cells. (a) Changes in cell survival by the combination of metformin/efavirenz/fluoxetine in HDFs and HCT116 cells. The cells were treated with metformin (5 mM), efavirenz (1.5 µM), fluoxetine (0.9 µM), metformin/efavirenz, metformin/fluoxetine, efavirenz/fluoxetine or metformin/efavirenz/fluoxetine combination for up to 96 h and their cell survival was assessed by the MTT assay. (b) Cellular ROS levels were measured by CellROX green staining in HDFs and HCT116 cells treated with metformin (5 mM), efavirenz (1.5 µM), fluoxetine (0.9 µM), metformin/efavirenz, metformin/fluoxetine, efavirenz/fluoxetine or metformin/efavirenz/fluoxetine combination for 24 h. (c) Cell cycle was analyzed with PI staining in HDFs and HCT116 cells treated with metformin (5 mM), efavirenz (1.5 µM), fluoxetine (0.9 µM), metformin/efavirenz, metformin/fluoxetine, efavirenz/fluoxetine or metformin/efavirenz/fluoxetine combination. The percentage of cell death was calculated by measuring G0 portion in histograms of cell cycle analysis (d) Changes in cell survival by the combination of metformin/efavirenz/fluoxetine combination in A549 and SK-Hep-1 cells. The cell survival was assessed by the MTT assay. Figure 2. When combination of metformin/efavirenz/fluoxetine was added to cultures of normal HDF cells and HCT116 cancer cells, the results of the follow-up MTT assay showed severe cell growth suppression and highly increased cellular ROS levels only in HCT116 cells but not in HDFs. Cell cycle analysis showed that cell death increased highly only in HCT116 cells due to amplification of cellular ROS.

When a combination of metformin/efavirenz/fluoxetine was added to cultures of normal HDF cells and HCT116 cancer cells, the results of the follow-up MTT assay showed severe cell growth suppression evident only in HCT116 cells but not in HDFs. Treatment with metformin alone, metformin/efavirenz, metformin/fluoxetine, or efavirenz/fluoxetine combination showed only a mild growth suppression of HCT116 cells but not HDFs (Figure 2a). As also expected from a previous report,^9^ cellular ROS levels were increased only in HCT116 cells but not in HDF when the cells were treated with metformin alone, metformin/efavirenz, metformin/fluoxetine, efavirenz/fluoxetine, or metformin/efavirenz/fluoxetine combination for 72 h (Figure 2b). Treatment with metformin/efavirenz/fluoxetine also showed the highest cellular ROS levels in HCT116 cells. Cell cycle analysis showed cellular ROS level-dependent cell death (% of G0 phase) only in HCT116 cancer cells. Such cell death was not observed in normal HDF cells. Treatment with metformin/efavirenz or metformin/fluoxetine combination showed a low percentage of cell death. Specifically, efavirenz/fluoxetine combination showed a 43% cell death as expected from cellular ROS level changes. Combination of metformin/efavirenz/fluoxetine recorded the highest cell death at 73.9% also expected by having the highest cellular ROS levels (Figure 2c). Addition of sodium acetyl cysteine (NAC), an ROS scavenger, completely blocked cell death in HCT116 cells, indicating that their cell death was due to their increased cellular ROS levels. A combination of metformin/efavirenz/fluoxetine showed a strong anticancer activity in other cancer cell types as well, such as in A549 human lung cancer cells, and SK-Hep-1 human liver cancer cells, suggesting that the observed cancer-cell-specific ROS amplification (CASRA) is applicable to multiple tumor types (Figure 2d).

### Combination of metformin/efavirenz/fluoxetine (M + E + F) induces ROS amplification via synergistic inhibition of mitochondrial membrane potential and mitochondrial electron transport complex I and III activities only in HCT116 cancer cells

**Figure 3.**
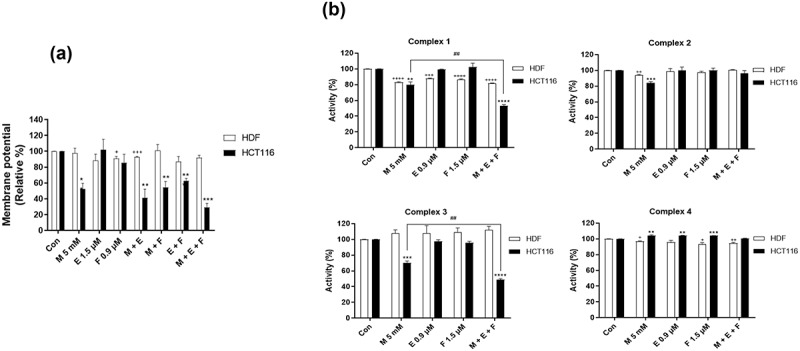
Effect of metformin, efavirenz, fluoxetine, metformin/efavirenz, metformin/fluoxetine, efavirenz/fluoxetine or metformin/efavirenz/fluoxetine combination on (a) mitochondrial membrane potential and (b) mitochondrial electron transport chain complex I, II, III, and IV activities in HDF and HCT116 cells. Mitochondrial membrane potential and mitochondrial electron transport chain complex I, II, III, and IV activities were measured in HDF and HCT116 cells treated with metformin (5 mM), efavirenz (1.5 µM), fluoxetine (0.9 µM), metformin/efavirenz, metformin/fluoxetine, efavirenz/fluoxetine or metformin/efavirenz/fluoxetine combination for 24 h. Vertical bars in the column graph indicate standard errors of the mean. Values are mean ± SEM (+p < .05, ++p < .01, +++p < .001, and ++++p < .0001 compared with the HDF control group; **p < .01, ***p < .001, ****p < .0001 compared with the HCT116 control group; ##p < .01 compared with the HCT116 M 5 mM group, n = 3). Figure 3. Combination of metformin/efavirenz/fluoxetine (M + E + F) induces ROS amplification via synergistic inhibition of mitochondrial membrane potential and mitochondrial electron transport complex I and III activities only in HCT116 cancer cells.

Cellular ROS levels were increased synergistically by a combination treatment with metformin/efavirenz/fluoxetine when compared to individual treatment with each drug or any combination of two drugs. There should be a reason for this synergistic increase in ROS levels. To pinpoint this reason, we measured changes in mitochondrial membrane potential, ATP concentration, and mitochondrial electron complex I, II, II, and IV activity after treatment with individual drug, or their combinations in HDFs and HCT116 cells for at 72 h post treatment. Their membrane potentials after treatment with an individual drug did not show any sizable differences in normal HDF cells (Figure 3a). However, the membrane potential after individual treatment with metformin and fluoxetine decreased by 50% and 10% each while efavirenz showed no change in HCT116 cancer cells. The combination treatment with metformin/efavirenz/fluoxetine reduced synergistically membrane potential by about 70% in HCT116 cancer cells (Figure 3a). The mitochondrial electron transport complex I and III decreased synergistically by about 50% by treatment with the three-drug combination of only in HCT116 cancer cells. There was no significant decrease in mitochondrial electron transport complex II and IV in either HDFs or HCT116 cells (Figure 3b). These results point to a synergistic increase in cellular ROS levels with the three-drug combination being possibly due to a synergistic decrease in activities of mitochondrial membrane potential and mitochondrial electron transport complexes I and III.

### Effect of drug combination M + E + F on the levels of DNA damage-induced apoptosis-, autophagy-, and necroptosis-related proteins in HCT116 and HDF cells

**Figure 4.**
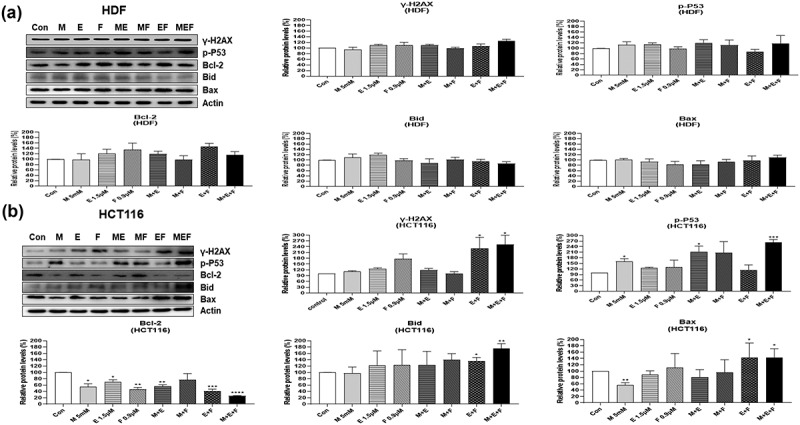
Changes in protein levels for DNA damage and apoptosis-related factors in HDFs and HCT116 cells treated with metformin/efavirenz/fluoxetine combination. After the cells were treated with metformin (5 mM), efavirenz (1.5 µM), fluoxetine (0.9 µM), metformin/efavirenz, metformin/fluoxetine, efavirenz/fluoxetine, or metformin/efavirenz/fluoxetine combination for 48 h, western blot analysis was performed to measure protein level changes for γ-H2AX, and p-p53 (Ser15), Bcl-2, Bid, and Bax. Statistical significance is indicated as *p < .05, **p < .01, ***p < .001, and ****p < .0001 compared with the control group (N = 3). Figure 4. Combination M + E + F increased the levels of DNA damage-induced apoptosis-related proteins only in HCT116 but not in HDF cells due to amplification of cellular ROS.

**Figure 5.**
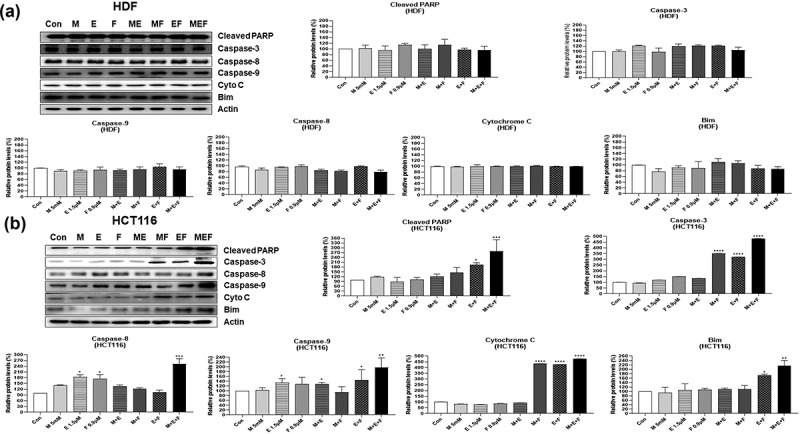
Changes in protein levels for apoptosis-related factors in HDF and HCT116 cells treated with the combination of metformin/efavirenz/fluoxetine. After the cells were treated with metformin (5 mM), efavirenz (1.5 µM), fluoxetine (0.9 µM), metformin/efavirenz, metformin/fluoxetine, efavirenz/fluoxetine, or metformin/efavirenz/fluoxetine combination for 48 h, western blot analysis was performed to measure the protein levels changes in cleaved-PARP, caspase-3, caspase-8, caspase-9, cytochrome C, and Bim. Statistical significance is indicated as *p < .05, **p < .01, ***p < .001, and ****p < .0001 compared with control group (N = 3).

**Figure 6.**
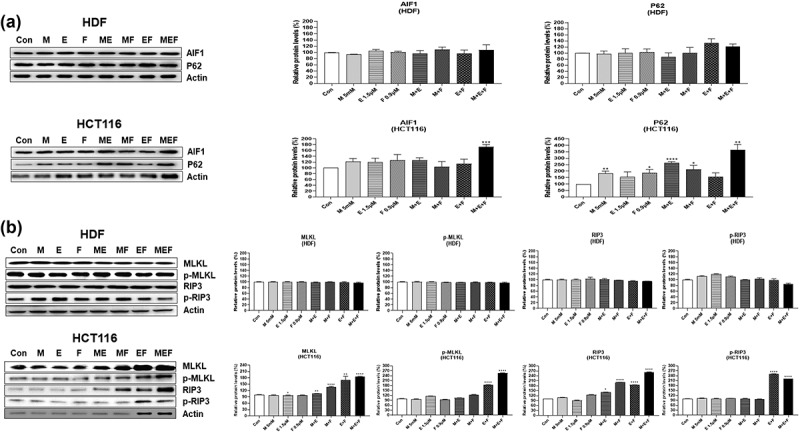
Changes in the protein levels for autophagy and necroptosis-related factors in HDFs and HCT116 cells treated with the combination of metformin/efavirenz/fluoxetine. After the cells were treated with metformin (5 mM), efavirenz (1.5 µM), fluoxetine (0.9 µM), metformin/efavirenz, metformin/fluoxetine, efavirenz/fluoxetine, or metformin/efavirenz/fluoxetine combination for 48 h, western blot analysis was performed to measure the protein levels changes in AIF1, p62, MLKL, p-MLKL, Rip3, and p-Rip3. Statistical significance is indicated as *p < .05, **p < .01, ***p < .001, and ****p < .0001 compared with control group (N = 3). Figure 6. Combination M + E + F increased the levels of DNA damage-induced autophagy- and necroptosis-related proteins only in HCT116 but not in HDF cells due to amplification of cellular ROS.

In an attempt to investigate the possible molecular mechanisms involved in cell death induced by combination M + E + F, expressions of several signal molecules were measured by western blot analysis in drug-treated HCT116 and HDF cells. The combination M + E + F increased the levels of γ-H2AX in HCT116 cells (*p* < .05), but not in HDFs, suggesting that severe DNA damage was induced by ROS amplification (Figure 4a-b). In addition, the combination M + E + F increased the levels of apoptosis-related proteins, p-p53, Bim, Bid, Bax, cleaved PARP, caspase 3, caspase 8, and caspase 9 expression in HCT116 cells (*p* < .05), but not HDFs (Figure 5a-b). Furthermore, the combination M + E + F increased cytochrome C expression, whereas it decreased the levels of Bcl-2 in HCT116 cells (*p* < .01) (Figures 4 and 5). However, the levels of cytochrome C and Bcl-2 expression were not altered in HDF cells (Figures 4 and 5). Finally, combination M + E + F increased the levels of AIF, P62, MLKL, p-MLKL, RIP3, and p-RIP3 in HCT116 cells (*p* < .05) but not in HDF cells (Figure 6a-b), suggesting that autophagy and necroptosis levels were also increased in the treated cancer cells (Figures 6a-b).

### Combination of metformin/efavirenz/fluoxetine (M + E + F) effectively reduces tumor growth in an in vivo model

**Figure 7.**
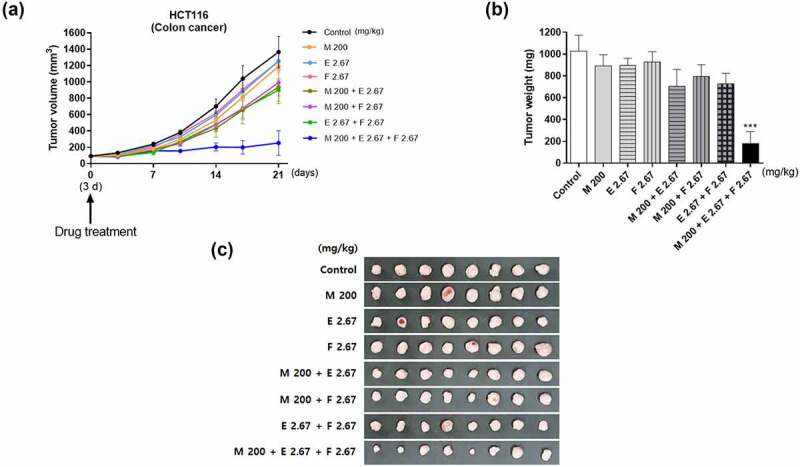
Effect of metformin, efavirenz, fluoxetine, metformin/efavirenz, metformin/fluoxetine, efavirenz/fluoxetine or metformin/efavirenz/fluoxetine combination on tumor volume and tumor weight increases in xenograft model of nude, athymic mice. The mice were injected subcutaneously with HCT116 (1 x 10^7^ cells in 100 μl) cells into their dorsal flank. When tumors reached a size of approximately 80 mm^3^, the mice were orally administered with metformin (200 mg/kg), efavirenz (2.67 mg/kg), fluoxetine (2.67 mg/kg), metformin/efavirenz, metformin/fluoxetine, efavirenz/fluoxetine or metformin/efavirenz/fluoxetine combination twice a day for 3 weeks. (a) Tumor volumes were measured as described in the Methods section. (b) The tumors were dissected and isolated from mice and the weight of each tumor was measured. (c) Photographs of the excised tumors were taken and are shown. Statistical significance is indicated as ***p < .001 compared with the control group (N = 8). Figure 7. An *in vivo* tumor model was prepared in female athymic nude mouse by injection of HCT116 colon cancer cells subcutaneously into the right flanks of each mouse. Oral treatment of metformin/efavirenz/fluoxetine (M + E + F) combination effectively reduces tumor growth in an *in vivo* tumor model.

To examine the *in vivo* anticancer activity of the combination of metformin/efavirenz/fluoxetine (M + E + F), HCT116 (1x10^7^ cells) cells were injected subcutaneously into the right flank of each mouse. On the seventh day post-cell implantation when the tumor sizes became about 80 mm^3^, each mouse was administered orally with 0.5% MC (methyl cellulose), metformin (200 mg/kg), efavirenz (2.67 mg/kg), fluoxetine (2.67 mg/kg), the combinations of metformin/efavirenz, metformin/fluoxetine, efavirenz/fluoxetine, or metformin/efavirenz/fluoxetine. All the drugs were orally administered twice a day for 3 weeks. The tumor sizes in the control group were about 1,400 mm^3^ after 3 weeks as shown in Figure 7a. Oral administration of metformin, efavirenz, fluoxetine, or the combination of metformin/efavirenz, metformin/fluoxetine, efavirenz/fluoxetine caused a slight reduction of tumor growth when compared to the control group. However, the tumor volume and tumor weight increases were significantly decreased in the mice orally fed with the combination of metformin/efavirenz/fluoxetine (*p* < .01) (Figure 7).

## Discussion

This study demonstrated that the combination of metformin/efavirenz/fluoxetine is a good example of anticancer treatment for colon cancer cells via cancer cell-specific ROS amplification (CASRA). The first such example of CASRA was the combination of metformin and apigenin, showing a profound anticancer activity in pancreatic cancer cells.^9^ Metformin first binds to mitochondria and causes an electron leakage, leading to the inhibition of mitochondrial respiration. Leaked electrons immediately react with surrounding H_2_O or O_2_ and generate ROS in both normal cells and cancer cells. Electron leakage in mitochondria by metformin results in a decrease in ATP levels only in normal cells but not in cancer cells as most of the cancer cell ATP is produced by the non-mitochondrial lactate fermentation, a phenotype described as the “Warburg effect” and the differences in energy metabolism in cancer cells and normal, non-transformed cells. However, targeting the Warburg induced glycolysis alone has not been very successful in cancer therapy.

Decreased ATP levels in normal cells induce AMPK activation (via phosphorylation at Thr172) and activated p-AMPK and then directly phosphorylates FOXO3a at Ser413, thus activating it. p-FOXO3a migrates to the nucleus as described in our previous report and brings about an increased transcription of MnSOD.^9^ The increased levels of MnSOD enzyme in normal cells efficiently remove cellular ROS in normal cells such as HDFs. However, in cancer cells, such as HCT116, ROS is not removed and ROS levels are relatively high because of the lack of AMPK/FOXO3a/MnSOD pathway contribution. The importance of contribution of FOXO3a/MnSOD to ROS production was confirmed by the results of ROS levels generated by treatment of HDFs with si-FOXO3a RNA and that being compared to transfection of wt-FOXO3a in HCT116 cancer cells.

Addition of efavirenz/fluoxetine to metformin further amplifies the ROS levels in cancer cells. Such cancer cell-specific ROS amplification (CASRA) leads to cell death in cancer cells via DNA damage-induced apoptosis, autophagy, and necroptosis as shown. HDF cells did not display increased ROS levels, DNA damage, or cell death. Efavirenz and fluoxetine are not simply ROS amplifiers. They also bind to mitochondria and inhibit their function. Each appears to cause electron leakage and induces a reduction of ATP production similar to metformin (data not shown). They also contribute to induction of AMPK/FOXO3a/MnSOD only in HDFs, as model normal cells. These compounds also generated cancer-cell-specific ROS amplification. Fluoxetine, a selective serotonin reuptake inhibitor (SSRI), is used as an antidepressant by modulating the levels of serotonin, and it is known to interact with mitochondria. It decreases State 3 respiration rates in isolated mitochondria, also reduces ATP synthesis rate and disrupts the phosphorylation potential of mitochondria. Moreover, fluoxetine inhibits the membrane-bound form of F1Fo-ATPase^42^ and decreases the function of mitochondria consuming complex I or complex II substrates.^43^ Efavirenz, an inhibitor of viral reverse transcriptase, also decreases mitochondrial membrane potential and enhances superoxide production,^44^ causing a concentration-dependent decrease in basal respiration and specifically in ATP production-coupled O_2_ consumption.^45^

Metformin, efavirenz, and fluoxetine each appear to interact with mitochondria and result in electron leakage from the mitochondrial membrane, thus decreasing ATP production and a reduction of mitochondrial potential and inhibition of mitochondrial electron transport chain complexes. The important point is that these compounds all induce mild electron leakage, cause mild inhibition of mitochondrial electron transport chain complexes, and mild reduction of ATP and mitochondrial membrane potential. They do not seriously affect cellular integrity and cell survival. However, certain chemicals such as potassium cyanide, doxorubicin, and arsenic cause severe mitochondrial damage and immediately induce cellular death.^46,47^ However, as the CASRA effect is sought, only mildly inhibiting compounds of mitochondrial membrane potential and mitochondrial electron transport chain complexes are suitable for being combined for amplifying ROS and inducing apoptosis only in cancer cells.

For metformin, efavirenz and fluoxetine, we observed a synergistic increase in cellular ROS levels in the combinational treatment with the three compounds when compared with their individual effects (Figure 2b). This synergistic amplification of ROS levels is the main mechanism in their strong anticancer activity. However, their mechanism for the synergistic increase in ROS levels is currently unclear. Inhibition of mitochondrial membrane potential and mitochondrial electron transport chain complexes by each individual drug treatment and by their combinational treatment provides a possible clue. Combinational treatment with the metformin/efavirenz/fluoxetine synergistically inhibits mitochondrial membrane potential and mitochondrial electron transport chain complexes I and III only in HCT116 cancer cells but not in normal HDF cells when compared with individual drug treatment (Figure 3b). This synergistic inhibition of mitochondrial membrane potential and mitochondrial electron transport chain complexes I and III explain possibly why cancer cells show a synergistic increase in ROS levels when treated with such a combination in cancer cells. We do not have an answer why the drug combination produces a synergistic inhibition of mitochondrial membrane potential and mitochondrial electron transport chain complex I and III only in cancer cells. Cancer cells may have a different structure of mitochondrial electron transport chain complex I and III to bind and inhibit more efficiently by a combination drug treatment. A more detailed experiment on mitochondrial structure and drug interaction to mitochondrial electron transport chain complex I and III will provide a better understanding of the mechanism.

**Figure 8.**
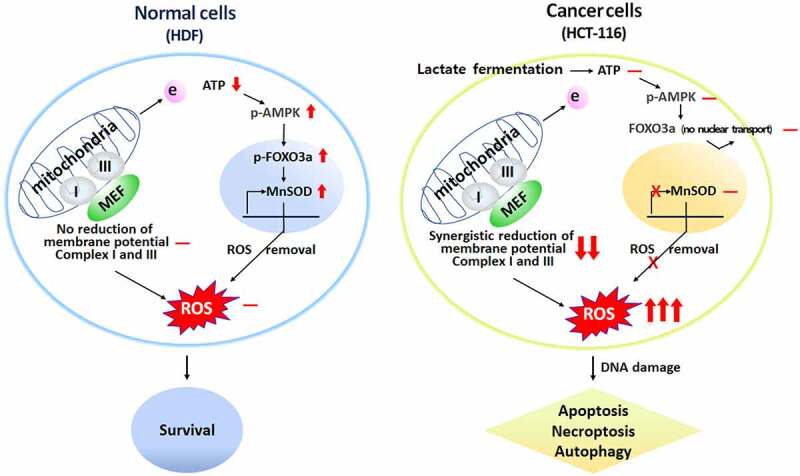
A hypothetical diagram depicting mechanism of cancer cell death induced via cancer cell specific ROS amplification by combination of metformin/efavirenz/fluoxetine.

Combinational treatment of metformin/efavirenz/fluoxetine inhibited up to 50% of mitochondrial electron transport chain complexes I and III in cancer cells but did not show any inhibition in normal cells. By comparison, doxorubicin inhibited mitochondrial electron transport chain complexes I by 70% in normal HDF cells and by 80% in HCT116 cells (data not shown). Doxorubicin is a general chemotherapeutic anticancer drug, displaying similar toxicity toward dividing normal cells and cancer cells. These results indicate that combinational treatment of metformin/efavirenz/fluoxetine combination displays a more selective anticancer activity than that from regular chemotherapeutic drugs. Activation of MnSOD via AMPK/FOXO3a/MnSOD pathway and subsequent removal of cellular ROS in normal cells is one of the main reasons for cancer cell-specific ROS amplification. Another reason is the cancer-cell-specific inhibition of mitochondrial membrane potential and mitochondrial electron transport chain complexes I and III by combinational treatment as pointed above. These two mechanisms may explain why ROS is synergistically amplified and induces cell death in cancer cell by the combination of these three compounds (Figure 8).

The results of the current study indicate that the cell death induced by metformin/efavirenz/fluoxetine combination is mediated through typical apoptosis, autophagy, and necroptosis. This is supported by the result that the combination of metformin/efavirenz/fluoxetine increases the levels of apoptosis-related proteins (such as H2AX, p-P53, Bid, and Bax) plus autophagy-related proteins (like AIF1, P62, and LC3B) in the treated cancer cells. Furthermore, we found that metformin/efavirenz/fluoxetine combination caused increases of necroptosis-related proteins (such as MLKL, p-MLKL, RIP3, and p-RIP3) in treated HCT116 cancer cells. However, with normal HDF cells, the protein levels related to apoptosis, autophagy, and necroptosis were not altered by the combination of these three compounds, suggesting that the anticancer activity induced by the combination of metformin/efavirenz/fluoxetine might be achieved via activation of apoptosis, autophagy, and necroptosis in cancer cells. It is generally well known that higher levels of ROS above a certain threshold cause irreversible DNA damage. This DNA damage might be responsible for the triggering of apoptosis, autophagy, and necroptosis revealed by the metformin/efavirenz/fluoxetine treatment in the cancer cells (Figure 8). The anticancer activity of the combination of metformin/efavirenz/fluoxetine was about the same compared to doxorubicin, which is known as a strong anticancer drug. Thus, it is suggested that the combination of metformin/efavirenz/fluoxetine can be considered as an anticancer drug candidate for clinical use.

An *in vivo* anticancer activity test using athymic, nude mice xenograft of human colon HCT116 cancer cells revealed that oral administration with metformin (200 mg/kg), efavirenz (2.67 mg/kg), and fluoxetine (2.67 mg/kg), respectively, slightly reduce growth of HCT116 xenografts if given alone. However, oral administration with the combination of metformin/efavirenz/fluoxetine caused a greater extent of the reduction of tumor volume increases, suggesting that the combination of metformin/efavirenz/fluoxetine exerts a profound anticancer effect in tumor growth. Administration with metformin alone causes a reduction in tumor growth in several animal cancer models.^48,49^ Although the antitumor activities of metformin, efavirenz, or fluoxetine alone appear not to be potent enough, the results of the present study suggest that the combination of metformin/efavirenz/fluoxetine is useful for cancer therapy and is a potential candidate drug combination for treatment of colon cancer. Further characterization of cancer-cell-specific ROS amplification and cell death in the tumor of in vivo cancer model by the combinational treatment of metformin/efavirenz/fluoxetine will provide a better understanding of their *in vivo* mechanism for anticancer activity.

In conclusion, we demonstrated here that a combination of metformin, efavirenz, and fluoxetine had strong anticancer activity in HCT116 human colon cancer cells via synergistic reduction of mitochondrial membrane potential and mitochondrial electron transport chain complex I and III through cancer cell-specific ROS amplification (CASRA). The apototic, autophagic, and necroptotic pathways play important roles in the production of anticancer activity of the combination of metformin/efavirenz/fluoxetine in cancer cells and *in vivo* nude mice test. Combination of metformin/efavirenz/fluoxetine caused dramatic reductions in tumor volume and weight in HCT116 xenograft model of nude mice. Our results indicate that combination of metformin/efavirenz/fluoxetine has profound anticancer activity both *in vitro* and *in vivo.*

### Cell culture and treat

The human cancer cell lines, HCT116 (colon cancer), A549 (lung cancer), Sk-Hep-1 (liver cancer), were obtained from the Korean Cell Line Bank (Seoul, Korea) and the HDF (human primary dermal fibroblast) were obtained from the Dermatology Laboratory of Seoul National University Medical School (Seoul, Korea). The cultured cells were grown in DMEM media with 10% FBS and in the presence of penicillin–streptomycin in a humidified incubator of 5% CO_2_ at 37°C. The cells were treated with metformin (TCI, M2009), efavirenz (TCI, E0997), fluoxetine (TCI, F0750), or doxorubicin (Merck, 25316-40-9).

### Transfection of cells with si-FOXO3a and wt-FOXO3a

HDF cells and HCT116 cells were grown to 70% confluence in a 100-mm dish and washed twice with 1× PBS. The cells were transfected with FOXO3a si-RNA (5’-UUGUUAGUCACUUUGCAUG-3’) (Cell Signaling) and wt-FOXO3a plasmid (Addgene, HA-FOXO3a WT) using lipofectamine 3000 transfection reagent (Invitrogen) according to the manufacturer’s instructions.

### MTT assays

The 3-(4,5-dimethylthiazol-2-yl)-2,5-diphenyltetrazolium bromide (MTT) (Merck Millipore, Burlington, MA) colorimetric assay was carried out according to the method by Mosmann.^50^ HDF, HCT116, A549, and SK-Hep-1 cells were cultured in 96-well culture plates (2 × 10^3^cells/well) for 24 h before drug treatment. Cell viability was measured up to 96 h by MTT assay using a microplate reader (Molecular Devices, SpectraMax Plus 384). Doxorubicin (2 μM) was used as a positive control in MTT assay.

### Cell cycle analysis

HCT116 and HDF cells were treated with metformin, efavirenz, or fluoxetine, a combination of metformin/efavirenz, metformin/fluoxetine, or efavirenz/fluoxetine, or a combination of metformin/efavirenz/fluoxetine for 48 hrs. The cells were washed with PBS, and fixed in cold 70% ethanol by adding cold ethanol and fixing at 4°C for 30 min while mixing slowly. After washing twice with PBS, the fixed cells were collected by centrifugation at 850 g. RNase A (ThermoFisher, DNase and protease-free) at 50 μl (100 μg/ml) was added to the cells and incubated for 30 min. Propidium iodide (Merck Millipore, P4170) at 200 μl (50 μg/ml) was added to the cells, and the cell cycle was analyzed using a flow cytometer (Becton Dickinson, Vantage SE cell sorter) and a mod Fit LT software (Verity Software, Topsham, ME). The percentage of cell death was calculated by measuring G0 portion in histograms of cell cycle analysis.

### ROS measurements

To measure cellular ROS levels in drug-treated HDF and HCT116 cancer cells, the cells were first washed with PBS. The cells were then incubated with CellROX Green Reagent (ThermoFisher, C10492) for 2 h and fixed with 4% paraformaldehyde. Photographs were taken using a fluorescence microscope (absorption 485 nm, emission 520 nm) (Zeiss, Axiovert 200). ROS intensity of each picture was quantified using Photoshop CS4 software (Adobe Systems). Cellular ROS level was calculated through dividing the ROS intensity by cell numbers in a picture and plotted as histograms.

### Measurements of mitochondrial membrane potential and mitochondrial electron transport chain complex I, II, III, and IV activities

Mitochondrial membrane potential was measured using tetramethyl-rhodamine ethyl ester (TMRE) kit (Cayman chemical, 701310). HDF and HCT116 cells were grown in 6-well culture plates and exposed to drugs for 72 h. The cells were trypsinized and collected by centrifugation at 250 *g*. The cells were then resuspended in 100 µl assay buffer and added 100 µl TMRE solution. The cells were incubated at room temperature for 30 minutes and collected by centrifugation at 250 *g* for 5 minutes. The cells were resuspended with 200 µl assay buffer. Membrane potential was measured using a flow cytometer (Becton Dickinson, Vantage SE cell sorter). To measure mitochondrial electron transport chain complex I, II, III, and IV activities, mitochondria were isolated from cultured cells using Mitochondria Isolation Kit (Abcam, 89874) according to the manufacturer’s protocol. The cells were washed with PBS and incubated on ice with reagent A. The cells were then homogenized and centrifuged at 1,000 *g* for 10 minutes at 4°C. The supernatant 1 was saved at −20°C, and the pellet was incubated for 10 minutes on ice with reagent B, homogenized and collected by centrifugation at 1,000 *g* for 10 minutes at 4°C. The supernatant 2 was saved. Supernatant 1 and supernatant 2 were pooled and centrifuged at 12,000 *g* for 15 min at 4°C. Their pellets were collected and resuspended in reagent C. Protein quantification was carried out by BCA assay. The final mitochondrial extract was saved at −80°C before use. The enzyme activities of the mitochondrial extracts were measured in triplicate with the following test kits: Complex I (Abcam, ab109721), Complex II (Abcam, ab109908), Complex III (BioVision, K520), and Complex IV (Abcam, ab109909). The mitochondrial extracts were loaded into each well at a concentration of 100 µg/200 µl (Complex I), 60 µg/50 µl (Complex II), 10 µg/200 µl (Complex III) or 20 µg/200 µl (Complex IV). A microplate reader (Molecular Devices, SpectraMax Plus 384) was used for the analysis.

### Western blot analysis

After twice washing with cold PBS, the drug-treated HDF or HCT116 cells were resuspended in the extraction buffer (150 mM NaCl/50 mM EDTA, pH 8, 1% Nonidet P-40) containing a mix of protease inhibitors (1 mM Phenylmethylsulfonyl fluoride, 5 μg/ml of aprotinin, 5 μg/ml of leupeptin). After centrifugation at 14,000 rpm for 30 min at 4°C, the cell lysates were isolated and used for western blot analysis. The protein concentration of the cell lysates was determined using the BSA assay reagent (BIOMAX, BCA0500). The cell lysates were resolved in 10% or 12% SDS-PAGE gels and electrophoretically transferred onto a PVDF membrane (Merck Millipore, IPVH00010). The membranes were blocked in 5% nonfat powder milk in TBST (50 mM Tris, pH 7.5, 150 mM NaCl, 0.1% Tween 20). The membranes were then incubated overnight with the proper dilution of primary antibody in milk/TBST and washed. The membrane was then incubated with diluted secondary antibody horseradish peroxidase conjugate (1:10,000) (ENZO, ADI-SAB-300-J), (Abcam, ab6728) for 2 h. The protein bands were visualized with the Immobilon reagent (Merck Millipore).

### In vivo test of anticancer activity

All animal experimental protocols were approved by the Laboratory Animal Committee of Hallym University (IACUC) (approval number Hallym 2021-34). Four-week-old female athymic nude mice (OrientBio, Seoul, Korea) were used in the present study. HCT116 cells (1 × 10^7^cells/100 μl) were subcutaneously injected into the right flanks of each mouse. On the seventh day after cell implantation for each mouse, the mice were administered with the following control or drugs: control (0.5% methyl cellulose), metformin (200 mg/kg), efavirenz (2.67 mg/kg), fluoxetine (2.67 mg/kg), metformin/efavirenz (200 and 2.67 mg/kg, respectively), metformin/fluoxetine (200 and 2.67 mg/kg, respectively), efavirenz/fluoxetine (each 2.67 mg/kg), or metformin/efavirenz/fluoxetine (200, 2.67, and 2.67 mg/kg, respectively) twice a day. Tumor volumes and body weights were measured twice a week. To gauge the tumor volume, a Vernier caliper was used. The tumor volume was calculated by the following formula: total volume = (length × width^2^)/2. After 21 days of treatment, the mice were sacrificed by cervical vertebral dislocation, and their tumors were dissected immediately and weighed. The dissected tumors were then stored in the deep freezer (−80°C).

### Statistical analysis

Statistical analysis was carried out by the Student’s t-test using GraphPad Prism Version 4.0 for Windows (GraphPad Software). P-values less than 0.05 were considered to indicate statistical significance. All values were expressed as mean ± S.E.M.

## Data Availability

All data generated or analyzed during this study are included in this article. The datasets used and/or analyzed during the current study are available from the corresponding authors on reasonable requests.
